# Macrophage Polarization: Learning to Manage It 2.0

**DOI:** 10.3390/ijms242417409

**Published:** 2023-12-12

**Authors:** Nadia Lampiasi

**Affiliations:** Istituto per la Ricerca e l’Innovazione Biomedica IRIB, Consiglio Nazionale delle Ricerche, Via Ugo La Malfa 153, 90146 Palermo, Italy; nadia.lampiasi@irib.cnr.it

The aim of this Special Issue is to investigate macrophages’ high plasticity and ability to differentiate/polarize in response to numerous stimuli in the context of diseases, infections, and biomolecules exposition (immunomodulators).

Macrophages can be categorized based on their ability to respond, produce, and secrete cytokines and active molecules (M0, M1, M2, M4). The naïve macrophages (M0) are found in tissues and contribute to cellular homeostasis, including the clearance of senescent cells, apoptotic bodies, and pathogenic materials [1]. M0 specialized macrophages are located in the brain (microglia), liver (Kupffer cells), lungs (alveolar macrophages), and bones (osteoclasts). When homeostasis is lost, naïve macrophages undergo polarization, stimulating different phenotypes such as M1 pro-inflammatory, M2 anti-inflammatory and M4 (Mox), an M1-M2 intermediate phenotype (Figure 1) [2].

Macrophages respond to bacterial infection, which triggers Toll-like receptors (TLRs), and to interferon-gamma (IFNϒ), which induces macrophage pro-inflammatory activation. Once activated, M1 macrophages release pro-inflammatory cytokines (Interleukin (IL)-1β, Tumor-Necrosis Factor- α (TNF)-α, IL-6), producing active molecules (reactive oxygen species (ROS), nitric oxide (NO)), and activate enzymes (cicloxygenase-2 (COX-2) and inducible nitric oxide synthase (iNOS)), which sustain inflammation [3]. Otherwise, macrophages respond to IL-4 and IL-13, inducing M2 polarization, which promotes cell proliferation and tissue repair [4]. Once activated, M2 macrophages can release various cytokines with different roles. Indeed, M2 macrophages are composed of plethora of different cell subtypes (M2a, M2b, M2c, M2d) that respond to many stimuli and can produce both pro- and anti-inflammatory cytokines. For instance, M2a macrophages remove cellular debris and contribute to tissue repair with the release of pro-fibrotic factors such as fibronectin, insulin-like growth factor (IGF), and transforming growth factor-β (TGF-β), but also can produce anti-inflammatory IL-10, and chemokines like chemokine ligand (CCL-17), CCL-18, and CCL-22, [5]. Meanwhile, M2b and M2c subtypes are known as regulatory macrophages because they contribute to the maintenance or restoration of cellular homeostasis. M2b macrophages are activated by IL-12, IL-23, and TNF-α and can release IL-1β, TNF-α, IL-6, and IL-10 [6]. On the other hand, M2c macrophages are activated by IL-10, TGF-β, or glucocorticoids, leading to increased release of IL-10, TGF-β, CCL-16, and CCL-18 [7]. Lastly, M2d subtype, which plays a role in wound healing [8], is activated by the adenosine 2 A receptor (A2AR), promoting the release of IL-10, vascular endothelial growth factor (VEGF), TNF-α, and IL-12. These latter macrophages have been identified in the ascites of ovarian cancer patients and can promote tumor progression [9]. Tumor-associated macrophages (TAMs) characterize the complexity of macrophages’ role in cancer. Indeed, TAMs are involved in the establishment of the tumor microenvironment, as they can act as both suppressive M1-like subtype and/or permissive M2d-like subtypes to aid tumor development. Current anti-cancer therapy is based on the possibility of transforming the permissive macrophages present inside the tumor into anti-tumor macrophages [10]. One manuscript in this Special Issue addresses this fascinating topic. More recently, an additional macrophage subtype was discovered and was named Mox or M4. This subtype possesses characteristics of both M1 and M2 phenotypes and undergoes polarization in response to oxidized low-density lipoprotein (LDL) or ROS. Like M1 macrophages, they produce ROS and NO, but they also display an enhanced capacity for phagocytosis, tissue remodeling, and tissue repair, like M2 macrophages [11]. Extracellular vesicles (EVs) are important for cell–cell communication. A recent study revealed that macrophages also use EVs to communicate, just as they use EVs produced by other cells (including tumor cells) to polarize into a specific subtype with the aim of protecting the organism [12]. Thus, two articles published in this Special Issue focus on EVs produced by parasites and tumor cells to polarize macrophages.

Macrophages are among the first cells to populate injured tissue, such as a transplanted organ [13], cancer tissues [14], or a damaged brain or heart [15,16]. This Special Issue sheds light on the molecular aspects of the specialization/polarization of macrophages in relation to certain diseases. One review included in this Special Issue investigates the relationship between inflammation and the non-genetic risk of developing autism spectrum disorder (ASD). Another manuscript focuses on bacterial infections and the mechanisms used to escape macrophages’ control, while others have studied the activation of signaling pathways (PNPLA7) involved in lipid-mediated macrophage polarization or immunomodulators which affect the polarization of macrophages (methotrexate, vitamin D). Finally, one manuscript characterizes the specialization of porcine macrophages, underlining the similarities and differences (few) between human and murine M1 and M2 macrophages.

Understanding the mechanisms and functional consequences of the phenotypic heterogeneity of macrophages will help us determine their potential role in diseases development. Furthermore, investigation on the macrophage’s plasticity could contribute to new therapeutic strategies.

I hope you will find these articles published in this Special Issue interesting, as they help to provide a clearer vision of the different properties of polarized macrophages.

## Figures and Tables

**Figure 1:**
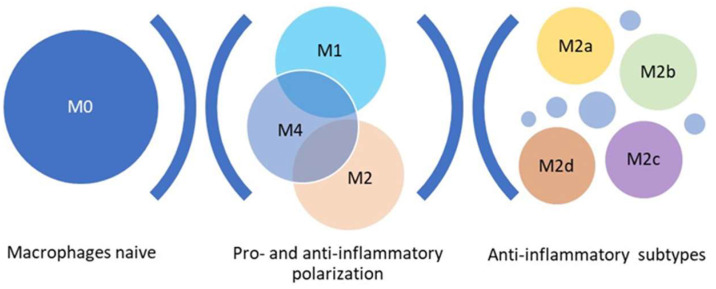
Schematic representation of the main steps of macrophage subtypes polarization. Small circles indicate intermediate subtypes located in solid tumors.

## Data Availability

Not applicable.
